# Access-related factors and e-cigarette use among 11–17-year-olds: a thematic synthesis of European studies using the five dimensions of access

**DOI:** 10.1186/s12889-026-26692-y

**Published:** 2026-02-17

**Authors:** Calum Lewis, Catherine Gallagher, Hannah Fairbrother, Duncan Gillespie

**Affiliations:** 1 School of Medicine and Population Health, University of Sheffield, Sheffield, UK; 2 School of Allied Health Professions, Pharmacy, Nursing and Midwifery, University of Sheffield, Sheffield, UK

**Keywords:** E-cigarettes, Vapes, Vaping, Youth, Policy, Adolescence, Nicotine, Access, Tobacco control

## Abstract

**Background:**

The rapid increase in e-cigarette use among young people is a public health concern that has led to new policies being discussed. This systematic review aimed to synthesise evidence on how access-related factors influence e-cigarette use among 11–17-year-olds in Europe, framing these factors using five dimensions of access (availability, affordability, accommodation, accessibility, acceptability) to inform thinking about policy approaches in the United Kingdom.

**Methods:**

A systematic literature search up to 31 March 2025 of studies published since 2016 was undertaken following PRISMA guidelines (PROSPERO: CRD42024614302). Studies focusing on e-cigarette access among 11–17-year-olds in European countries were eligible for inclusion. We searched EMBASE, PsycINFO, and Web of Science. Findings were narratively synthesised using a Joanna Briggs Institute (JBI) convergent integrated approach; study quality was assessed using JBI checklists. Findings were synthesised thematically using the five dimensions of access as an organising framework.

**Results:**

Twenty-one studies met the inclusion criteria, with some relevant to more than one dimension of access. Nine studies referred to accommodation, with a focus on how key spaces such as schools could limit the ease of e-cigarette use, a challenge complicated by the inherent concealability of e-cigarettes. Ten studies referred to acceptability, as influenced by restrictions on industry promotion of e-cigarettes to young people. Six studies referred to accessibility, which highlighted a particular challenge of age verification at the point of purchase. Five studies referred to affordability, emphasising the importance of relatively low prices as a key marketing tool to young people. Three studies referred to availability, highlighting access via social networks as a key way of circumventing other restrictions. However, there was a lack of evidence on how changes to the different dimensions of access might interact to modify the effects of new regulation.

**Conclusion:**

The five dimensions of access are a potentially useful way to structure thinking about how new regulation might affect young people’s e-cigarette use. The synthesis highlights that young people’s vaping behaviour operates in a complex system of interacting factors; and that evidence gaps remain in developing a clearer picture of this system, including interactions.

**Supplementary Information:**

The online version contains supplementary material available at 10.1186/s12889-026-26692-y.

## Introduction

Nicotine containing e-cigarettes (vapes) have become popular in many countries as an effective aid in quitting tobacco use [1–3]. However, since around 2021, vaping has rapidly risen in popularity among people who have never used tobacco, particularly young people [4, 5]. For example, as of 2024, approximately 18% of young people aged 11–17 in Great Britain reported having tried an e-cigarette, up from 13.9% in 2020 [6]. This trend has created a dilemma for public health policymakers in how to protect young people from the potential physical and mental health harms of regular vaping [7].

Current evidence on the physical health harms of vaping in people who have never smoked is limited. While long-term data on specific disease outcomes remain scarce, some studies report associations with biomarkers or intermediate outcomes that could be relevant to cardiovascular disease [8], cancer [9], and respiratory illness [10]. Given these potential links, there have been calls for further long-term research on cardiovascular effects and biomarker changes [9, 11, 12]. Alongside potential physical health effects, there is evidence that nicotine addiction may adversely affect young people’s mental health and brain development, with possible longer-term cognitive impacts [13].

Therefore, despite consensus that vaping is much less harmful than tobacco smoking [11], concerns about nicotine addiction and uncertainties regarding other long-term effects have driven the United Kingdom (UK) Government’s introduction of new regulatory measures [14–16].

Central to these interventions is the concept of access to vapes. Access can be defined as having five dimensions: availability, affordability, accommodation, accessibility and acceptability [17]. These dimensions provide a structure for understanding the factors that vaping regulations aim to influence. Each dimension is a potential target for intervention, and a single intervention may affect multiple dimensions. Furthermore, interactions between dimensions may modify the effects of regulation on vaping behaviour. For example, in June 2025, the UK Government reduced the availability of disposable vapes (reported to be popular among young people [14]) by banning them. These effects may be amplified by the introduction of the planned new tax on e-liquids, which aims to reduce the general affordability of vaping [15].

Although originally developed to describe use of healthcare services [17], the dimensions of access are well suited to this review because the structural [16], social [18], and market-related [4] factors they capture can also influence young people’s e-cigarette use. In this review, the framework is used to structure and synthesise evidence on how these factors influence patterns of young people’s e-cigarettes use. The framework is not used to test causal mechanisms or to evaluate the validity of the access dimensions themselves, but rather to support synthesis and to map findings to potential policy levers.

The aim of this review is to synthesise evidence on how access-related factors influence e-cigarette use among 11–17-year-olds; the typical age range of secondary education. Evidence is synthesised thematically the using five dimensions of access to support interpretation of the European evidence base in relation to the UK context. Given the limited availability of UK-specific evidence, this review focused on studies conducted within Europe, reflecting some shared regulatory frameworks established under the Tobacco Products Directive (TPD) [19, 20]. Important differences in market characteristics and social contexts between Europe and the UK are acknowledged and considered in the Discussion.

## Methods

### Definition of the five dimensions of access

Working definitions of the five dimensions of access were developed to describe young people’s access to e-cigarettes using the definitions of Penchansky and Thomas [17]. The definitions were then mapped to the e-cigarette context and refined in the pilot phase of the review; final definitions are presented in Table 1.

**Table 1:** Definitions of dimensions of access

Dimension of access	Penchansky & Thomas (1981) Definition	Review Definition
Affordability	The relationship between the price of services and the client’s ability to pay, including indirect costs such as transportation and lost wages.	The price of e-cigarettes in relation to a young person’s ability to pay. This includes the direct cost of products, such as cheap disposable vapes, and the relative cost compared to alternatives like tobacco cigarettes. Affordability is influenced by policies such as taxation, which directly target product price, and restrictions on availability of certain products that may also be low-cost options.
Acceptability	The relationship between clients’ attitudes about personal and practice characteristics of providers and providers’ attitudes about acceptable personal characteristics of clients. This includes issues such as age, sex, ethnicity, and social status.	How well-aligned e-cigarettes are with young people’s personal preferences and social norms, and how this alignment influences their decision to use them. This could include social desirability as influenced by perceptions of peer use, and perceptions of the health harms of e-cigarettes. Industry primarily influences acceptability through advertising, including point-of-sale displays and online promotions.
Accommodation	The manner in which the supply of healthcare services is organised to accept clients, including appointment systems, hours of operation, and telephone services.	How easily young people can use e-cigarettes in various settings. This is influenced by policies creating vape-free spaces (e.g., in schools, on public transport) and the degree to which people are aware of these rules and adhere to them. It is also affected by inherent product characteristics, such as device size and minimal odour, which enable discreet use in prohibited areas.
Availability	The volume and type of existing services and resources in relation to clients’ needs. This includes supply factors such as sufficient personnel, facilities, and equipment.	The overall supply of e-cigarettes accessible to young people through both formal and informal channels. This is influenced not only by market-level legislation like product bans (e.g., on disposables) or increases in the Minimum Legal Sales Age, but also by informal social networks, such as sourcing from older siblings, friends, or peers.
Accessibility	The relationship between the location of supply and the location of clients, considering travel time, distance, and transportation options.	The ease with which young people can obtain e-cigarettes from commercial retail sources. This includes the density and proximity of physical retailers (e.g., vape shops, convenience stores), which can be influenced by policy measures like retailer licensing schemes, and the ease of purchasing through online channels. It is significantly influenced by retailer practices, such as the enforcement (or lack thereof) of age-of-sale verification at the point of purchase.

### Search strategy

A systematic literature search was conducted to identify studies examining young people’s access to e-cigarettes within European countries, following Preferred Reporting Items for Systematic reviews and Meta-Analyses (PRISMA) guidelines. The protocol and any amendments can be found on Prospero (CRD42024614302). The search covered three databases, EMBASE, PsycINFO, and Social Sciences Citation Index (Web of Science), chosen for their disciplinary coverage of health, addiction, and social sciences. The search was completed using a combination of keywords and MeSH terms related to each dimension of e-cigarette access (Table 1), young people, and policy measures. Search strings were developed iteratively, incorporating terms such as “e-cigarette*”, “young person” and “accessibility” (see Supplementary Methods). The search was supplemented by searching reference lists of relevant studies and reports to identify additional sources. Studies published before 2016 were not included to ensure that the focus of the review was on current and emerging trends, such as the growth in disposable products [4, 6]. This time point was selected due to the implementation of the TPD in 2016, given it resulted in changes to regulatory frameworks [19, 20]. The final searches of each database were on 31/03/2025. This review focused on peer-reviewed empirical studies. Grey literature was not searched or included as part of the evidence synthesis. Titles and abstracts were screened by one reviewer (CL) to identify potentially relevant records. To support consistency in screening decisions, a second reviewer (CG) independently checked a subset (15%) of records at the title and abstract stage. The remaining records were then assessed for eligibility at full-text by CL. A subset of full-text assessments (15%) was subsequently checked by CG to ensure consistency. Agreement between reviewers was high (91%) across the subset checks. The final list of included studies was compiled by CL, with any borderline cases discussed and resolved through discussion with CL, DG, and HF.

### Inclusion criteria

Studies were eligible for inclusion if they met the following criteria: (1) peer-reviewed primary research employing qualitative, quantitative, or mixed-methods designs; (2) focused on individuals aged 11–17; (3) examined any of the five dimensions of e-cigarette access (Table 1); (4) were conducted at least partly in European countries; (5) reported on the relationship between access and e-cigarette usage; (6) were published from 2016 onwards; and (7) were published in English or with an English language translation. The focus on European studies reflects shared regulatory frameworks relevant to the UK, including legislation developed during the UK’s membership of the European Union, such as the TPD [19, 20].

### Assessment of study quality

The methodological quality of included studies was assessed using the Joanna Briggs Institute (JBI) Critical Appraisal Tools [21], which are suitable for evaluating the range of study designs included in the review. Studies were appraised across domains such as sampling strategy, risk of bias, appropriateness of statistical methods, and relevance of findings (see Supplementary Results). One reviewer (CL) conducted the assessments, with 15% reviewed independently by two further reviewers (HF, DG), with any disagreements resolved through discussion. No studies were excluded based on quality, and no formal weighting or prioritisation was applied. Instead, quality appraisal informed how findings from individual studies were interpreted within the synthesis. Findings from studies assessed as lower-quality were interpreted more cautiously and were not used on their own to support key interpretive claims but were considered in conjunction with evidence from higher-quality studies.

### Data synthesis

The JBI convergent integrated approach [22] was used to synthesise data across diverse study designs. This methodology allowed the integration of quantitative studies (e.g., prevalence and initiation rates) with qualitative research on behavioural and contextual factors influencing access. Quantitative findings were transformed into narrative statements describing the direction and nature of reported relationships, rather than being statistically pooled, to allow integration with qualitative findings. This process is referred to as ‘qualitisation’ in mixed-methods synthesis [22], and aligns with established guidance on narrative synthesis [23] and the JBI Manual for Evidence Synthesis [22]. The ‘qualitised’ and qualitative data were then organised using the five dimensions of access as an organising framework, with findings mapped to relevant policy areas. Results were interpreted in the broader policy and cultural context of the UK and the European countries on which the studies were based. Study quality informed interpretation during synthesis, as described in the quality appraisal section. The results of the synthesis are structured by the five dimensions of access.

## Results

### Search results

**Fig. 1:**
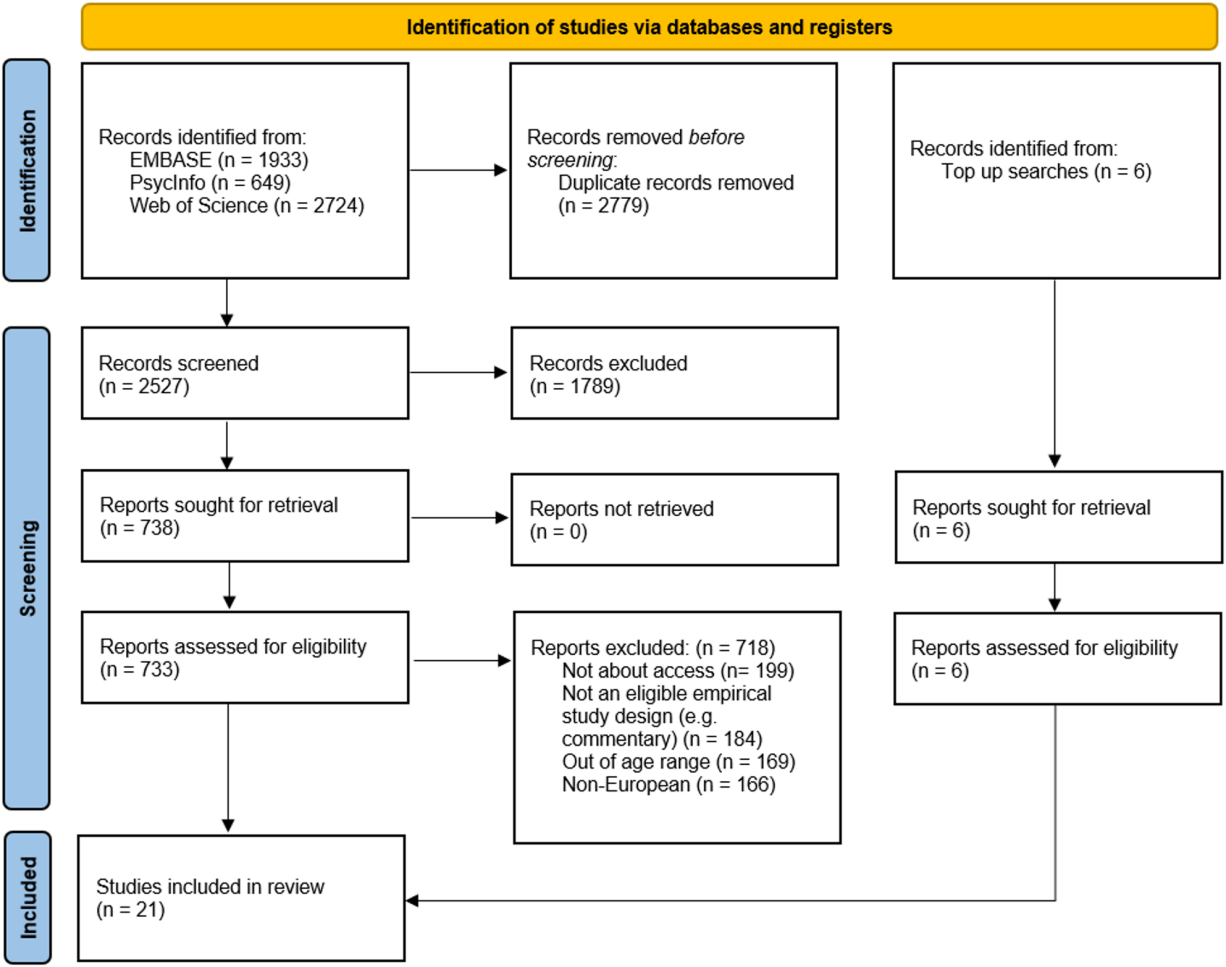
PRISMA Flow Diagram showing study identification

The initial search across three databases yielded a total of 5,306 records. After removing 2,779 duplicates using the Jaro-Winkler method of matching titles [24], 2,527 unique records remained for screening. Title and abstract screening led to the exclusion of 1,789 records that did not meet the review’s scope or preliminary inclusion criteria. The full texts of the remaining 738 potentially eligible articles were retrieved and assessed against the pre-defined inclusion and exclusion criteria. During this full-text review, 718 studies were excluded. At full-text review, the most common reasons for exclusion were: studies that did not examine access-related factors relevant to e-cigarette use; studies that did not focus on the target age range; studies that did not report primary empirical data on e-cigarette use; and studies conducted outside the predefined geographical scope. The final synthesis included 21 studies, made up of 15 studies from the initial search and 6 further studies that were identified through top-up searches to present a view of the literature up to 31/03/2025. The detailed flow of study identification, screening, eligibility assessment, and inclusion is presented in the PRISMA flow diagram (Fig. 1).

### Study characteristics

**Fig. 2:**
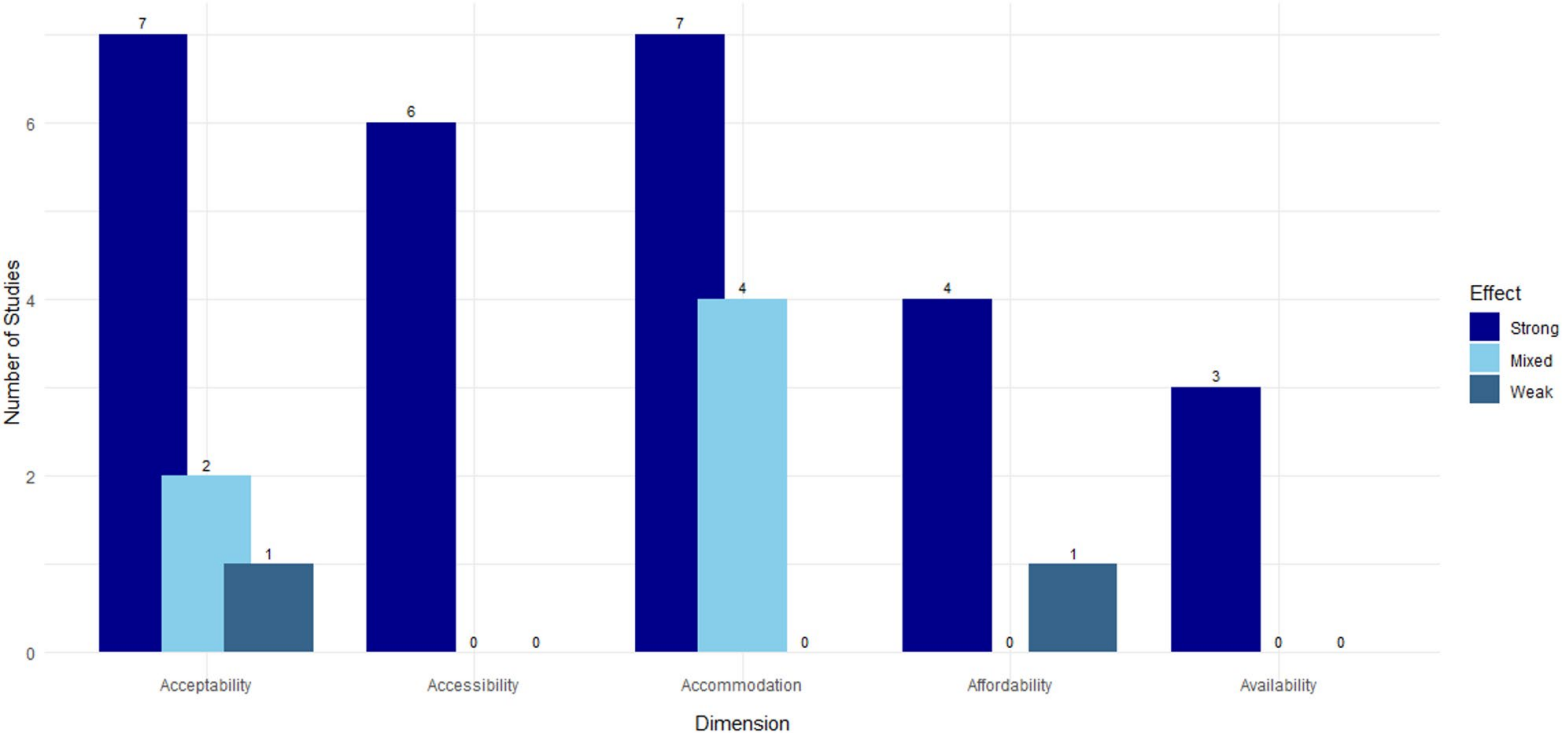
Number of Studies by Access Dimension and Effect Size Strong: Study reports clear, consistently meaningful effects of the access dimension Mixed: Study findings on the access dimension are inconsistent or contradictory Weak: Study finds no discernible effect

The included studies were published between 14/04/2016 and 05/03/2025. Key characteristics of the included studies are summarised in Table 2. The geographical focus of the research was predominantly the UK, which was the setting for eleven studies [25–35]. Other studies were conducted across various settings, including Europe (one multi-country study) [36], worldwide (three multi-country studies) [37–39], Germany [40], Albania [41], Poland [42], the Netherlands [43], Norway [44], and one study encompassing Denmark, Finland, and Norway [45]. Of the total of 21 studies, 14 provided quantitative data and 7 provided qualitative data. The methodological quality of the included studies assessed using the relevant JBI critical appraisal checklist ranged from “Low-Moderate” to “High” (see Table 2 and Supplementary Results). Common limitations that factored into the quality appraisal were a reliance on self-reported measures, cross-sectional designs, and limited adjustment for confounding. In Fig. 2, studies have been categorised by access dimension and whether the study indicates there is evidence that the effect of the dimension on e-cigarette usage is strong, mixed or weak (see Fig. 2).

**Table 2:** Key characteristics of included studies

Reference Number	Author (Year)	Country	Study design	No# of Participants	Age	Data source / method	Overall quality
[24]	Weishaar et al. (2016)	UK	Qualitative	83	14–17	Focus groups	Moderate–high
[25]	Notley et al. (2025)	UK	Qualitative	29	16–20	Group and individual interviews	Moderate–high
[26]	Smith et al. (2023)	UK	Qualitative	82	11–16	Focus groups	Moderate–high
[27]	Williams et al. (2023)	UK	Cross-sectional survey	2,613	11–18	Online survey	High
[28]	Moore et al. (2023)	UK	Mixed-method natural experiment evaluation	360,000 (quantitative); 221 (qualitative)	13–15	In-person survey Qualitative interviews and retail observations	High
[29]	Smith et al. (2025)	UK	Qualitative	33	12–16	Co-produced classroom activities and focus groups	Moderate–high
[30]	De Andrade et al. (2016)	UK	Qualitative	182	13–16	Co-produced classroom activities and interviews	Moderate–high
[31]	Parnham et al. (2016)	UK	Cross-sectional survey	12,445	11–18	Online survey	High
[32]	Kirkcaldy et al. (2019)	UK	Qualitative	64	11–17	Group interviews	Moderate–high
[33]	Hallingberg et al. (2016)	UK	Cross-sectional survey	7,376	11–16	In-person survey	High
[34]	Best et al. (2016)	UK	Cross-sectional survey	3,808	11–19	In-person survey	High
[35]	Cerrai et al. (2022)	Europe	Cross-sectional survey	99,648	15–16	In-person survey	High
[36]	Ylitörmänen et al. (2024)	Worldwide	Cross-sectional survey	165,299	11–17	In-person survey	High
[37]	Chan et al. (2022)	Worldwide	Cross-sectional survey	151,960	13–15	In-person survey	High
[38]	Braak et al. (2020)	Worldwide	Cross-sectional survey	12,128	16–19	Online survey	Moderate–high
[39]	Hansen et al. (2020)	Germany	Longitudinal cohort	4,529	10–16	In-person survey	Moderate–high
[40]	Dadras (2024)	Albania	Cross-sectional survey	9,985	13–15	In-person survey	Moderate–high
[41]	Balwicki et al. (2018)	Poland	Cross-sectional survey	341	16–17	In-person survey	Low-moderate
[42]	Rozema et al. (2018)	Netherlands	Quasi-experimental longitudinal study	7,733	11–18	In-person survey	Moderate–high
[43]	Scheffels et al. (2023)	Denmark, Finland, Norway	Qualitative	46	15–20	Focus groups	High
[44]	Tokle (2020)	Norway	Qualitative longitudinal study	118	12–17	Group and individual interviews	High

## Results of synthesis

### Acceptability

Acceptability (see Table 1) refers to how well-aligned young people’s perceptions of e-cigarettes are with their personal, social and harm preferences. The studies indicate the strong influence of industry promotion of e-cigarettes to young people, with point-of-sale advertising in small shops and internet advertising playing key roles in shaping perceptions and potential usage [28, 35, 40, 41]. For example, one UK study showed that advertising increased e-cigarette uptake, with a 17-year-old explicitly mentioning that promotional content had influenced his decision to try e-cigarettes [25]. In another UK study, the participants reported that promotions were ‘unavoidable’ and that they were aimed at young people [26]. Whilst some young people said that the packaging did not influence their decisions, many said that it did influence their usage [26]. Another UK-based study [30] found a less clear relationship between advertising and use, with young people expressing mixed views on whether e-cigarette advertising influenced their behaviour, although they noted that the strategic placement of advertisements, particularly near shop tills, were difficult to ignore. Some participants stated that advertisements made them curious about trying e-cigarettes, while others believed that advertising alone was not enough to encourage use [30]. Participants often pointed to peer influence as a stronger factor in shaping e-cigarette use, noting that seeing friends vape was more impactful than advertising itself [30].

While some studies suggest that advertising bans may reduce young people’s e-cigarette use, the evidence remains mixed, with variations across regulatory contexts. Two studies assessed the impact of advertising bans at a country-level. One study, using data from the 2019 European School Survey Project on Alcohol and Other Drugs, found that countries with stronger advertising bans had a 21% lower likelihood of young person e-cigarette use [36]. However, a cross-sectional study using data from the Global Youth Tobacco Survey (2016–2019) and WHO Framework Convention on Tobacco Control (FCTC) implementation reports found no significant association between the implementation of WHO FCTC Article 13 advertising bans and young people’s e-cigarette use in high and upper middle-income countries [37]. Further evidence on the relationship between advertising regulations and e-cigarette usage comes from a natural experiment evaluating the effects of the EU Tobacco Products Directive (TPD) [29]. This evaluation found that self-reported exposure to e-cigarette advertising declined following implementation of the TPD, alongside a stalling or slight decline in previously increasing rates of e-cigarette use among young people. However, there is an issue when attributing this decline to the advertising restrictions brought in by the TPD, as it was part of another change that involved warnings on product labels.

None of the included European studies examined how exposure to promotion via social media or influences affected young people’s use.

### Affordability

Affordability (Table 1) relates to the price of e-cigarettes in relation to a young person’s ability to pay. Research in the UK consistently highlights that young people view e-cigarettes favourably due to their low price [26, 27, 31]. MJ Smith, AM MacKintosh, A Ford and S Hilton [27] highlighted that young people viewed disposables as “cheap and cheerful”, which could be an attractive factor for initiation [27]. Similar perspectives were shared in a Scottish school-based study where students perceived e-cigarettes as more affordable than combustible cigarettes, with one pupil noting they spent significantly less money compared to purchasing cigarettes [31]. A more recent UK-based qualitative study also supports the role of affordability, particularly for disposables [26]; many participants noted they were “cheap” and easy to purchase, though some mentioned the total cost could increase over time if addiction led to more frequent purchases [26].

While qualitative evidence highlighted the importance of low e-cigarette prices, this review found no quantitative European studies that assessed the direct effect of e-cigarette price changes on demand among young people. The quantitative evidence that was identified focused instead on the impact of relative pricing (i.e., cigarette prices) on vaping prevalence amongst young people, which was limited to two studies with conflicting findings. One study [38] found evidence consistent with substitution, where higher cigarette taxation was significantly associated with significantly higher odds of vaping [38]. In contrast, the other study [36] found young people in countries with higher cigarette prices had significantly lower odds of current e-cigarette use [36].

### Accessibility

Accessibility (Table 1) concerns the ease with which young people can obtain e-cigarettes from commercial sources. The literature suggests that young people exploit perceived weaknesses in age verification and enforcement, primarily through smaller physical retailers and online platforms [26, 27, 31, 32]. Research consistently finds e-cigarettes are perceived as easy to obtain, with corner shops frequently cited as popular locations due to perceptions of weak enforcement of age restrictions compared to supermarkets or dedicated vape shops [26, 27, 31]. Young people report sharing knowledge about lenient stores and easily bypassing age verification both in these shops and on online platforms [26, 27, 31].

Quantitative data highlights the differing routes young people can use to purchase e-cigarettes. One high-quality UK study tracked a significant shift between 2019 and 2022, showing increased odds of purchase from small shops (including vape and corner shops) and decreased odds of purchasing online among current young person users [32]. This study also found young people aged 14–17 were significantly less likely than 18-year-olds to source from supermarkets [32]. Despite varied definitions of retail outlets in studies [32, 39], a consistent finding emerges: smaller retailers are key access points for young people [26, 27, 31, 32, 39]. Furthermore, purchasing behaviours can differ by setting; a study in Poland highlighted greater online purchasing among rural young people, suggesting context influences on access routes [42].

### Accommodation

Accommodation (Table 1) relates to how easily young people can use e-cigarettes in various settings. The evidence suggests that the inherent concealability of many e-cigarettes poses a significant challenge to the effective implementation of use restrictions in spaces like schools. There is evidence that young people find e-cigarettes easier to conceal compared to conventional cigarettes due to their size and lack of persistent smoke or odour, enabling use on school premises, buses, and bedrooms [25–27, 31, 33, 44, 45]. The literature indicates that ease of concealment and perceived weak enforcement allow young people to circumvent existing rules [25, 26]. This practice, sometimes termed “stealth vaping” [26], is further enabled by perceptions that school rules are not always strictly enforced [25]. Some accounts suggest that punishments, when applied, are ineffective deterrents and may add to the transgression’s appeal [26].

Quantitative studies also find limited effects of restrictions. A national survey in Wales reported no statistically significant association between the perceived strength of school policies and student e-cigarette use [34], while an experimental study evaluating an outdoor school grounds ban found it did not significantly alter the trajectory of student e-cigarette use compared to control schools [43]. These findings suggest that school-level policies on vape-free spaces are ineffective, potentially meaning that similar policies would also not have the desired effect if translated to other public spaces.

### Availability

Availability (Table 1) concerns the overall supply of e-cigarettes accessible to young people through both formal and informal channels. Research highlights the significant role of social connections in how young people obtain e-cigarettes, demonstrating availability beyond commercial retail. Qualitative studies reveal that young people frequently source e-cigarettes indirectly through older siblings or friends who meet the legal purchase age [25, 45]. Furthermore, school environments can function as informal distribution hubs, with older students sometimes selling products to younger peers [26, 45]. This peer-to-peer supply bypasses retail regulations, meaning availability is not solely determined by the presence or practices of shops but is embedded within young people’s social networks [26, 45].

Recognising these dual pathways (retail and social) is crucial when considering interventions targeting availability. Despite the evidence indicating that young people’s access to e-cigarettes is shaped by informal supply chains, this review found no quantitative European studies on the effect of raising the Minimum Legal Sales Age (MLSA) for e-cigarettes, which would reduce the number of legal purchasers in social circles. Similarly, there was limited evidence on the direct impacts of banning disposable vapes (which would eliminate the legal supply). This is particularly relevant given that qualitative evidence has noted that young people themselves perceive disposables as a “young person’s product” [26], suggesting their popularity. The research also raises concerns about the potential unintended consequences of such bans, including users stockpiling, seeking illicit sources, switching to other vape types like refillables, or possibly initiating or returning to traditional cigarette use [26].

## Discussion

The aim of this review was to synthesise evidence on how access-related factors influence e-cigarette use among 11–17-year-olds to inform thinking about regulatory approaches in the UK. The discussion interprets patterns in the evidence rather than evaluating or predicting the effects of specific policies. Our findings show how the five dimensions of access to e-cigarettes relate to young people’s behaviour, and how different types of interventions map onto these dimensions. However, there was no evidence in the literature on how the different dimensions of access interact within a complex system to shape young people’s vaping behaviours.

It is well established that e-cigarette use amongst young people has been driven by young people’s perceptions of the widespread use of e-cigarettes, ease of purchase, low cost, peer influence, product promotion and knowledge gaps regarding health harms [7, 18, 46]. By adapting the five dimensions of access to the context of e-cigarette use by young people, this review provides a method to categorise these previously identified drivers. For example, peer influence can be understood as a factor relating to both social availability and acceptability, while product promotion and perceptions of harm primarily affect acceptability.

This categorisation can support more systems-based thinking about developing coordinated policy strategies that intervene across multiple dimensions of young people’s access to e-cigarettes [47]. Decomposing access into its five interlinked dimensions is helpful because it allows different policy targets to be identified and highlights how interventions in one area may influence others, including through unintended consequences [48, 49]. For example, price rises or age of sale restrictions may target the affordability and accessibility of e-cigarettes to young people. If these measures reduced young people vaping, they may also indirectly limit availability through smaller social supply networks [18]. However, if these interventions are ineffective (e.g. through proxy purchasing by adults) then policies that reduce the acceptability of vaping among young people, and their accommodation to do so, may become more important [18]. It is also important to consider young people’s access to unregulated e-cigarettes within this systems perspective. Young people may not be aware that they are using an unregulated product, due to the visual similarity to regulated products [7], but unregulated products may pose higher health harms [7].

The main strength of this review is its comprehensive search across varied literature and the synthesis of findings using the five dimensions of young people’s e-cigarette access. However, several key limitations of the review must be acknowledged.

First, the breadth of the review, while a strength, limited the depth of analysis regarding the specific behavioural determinants underpinning each dimension of e-cigarette access. Future research could deepen these findings by applying behaviour change theories like the COM-B model to explore these determinants in a more granular way [50].

Second, title and abstract screening was conducted by a single reviewer, which may have increased the risk of missed studies. The risk was mitigated through broad search strategies, subset checking by a second reviewer, and discussion of borderline cases.

Third, the review does not address policies affecting the supply of unregulated vapes, nor does it address policies regulating product content such as flavour or nicotine dose. Regulations on these product-specific factors could affect the value that young people place on e-cigarettes, and therefore their purchasing behaviour [51]. These other interventions are critical components of a comprehensive public health strategy to address young people’s vaping [7].

Fourth, the five dimensions of access, by their nature, focus on upstream, structural, and population-level policy approaches rather than individual-level interventions focused on education or cessation support for young people who vape [52].

Despite these limitations, focusing on upstream factors that shape young people’s access to products remains important within broader efforts to address health inequalities [53]. This is particularly relevant to e-cigarette use in the UK, given that data has shown an association between socioeconomic disadvantage and vaping among never-smoking young people [54].

Finally, a further limitation of this review relates to heterogeneity operating at two levels: within Europe, and between European and other global contexts. While focusing on European studies was intended to maintain relevance to the UK, given some shared legislation [19, 20], substantial variation exists across European countries in regulatory, market, and social contexts. Regulatory differences include variation in advertising restrictions; market differences include the availability of disposable e-cigarettes and alternative nicotine products; and social differences include norms around vaping and informal supply through peers. For example, snus is widely used in Sweden [55], but banned in the UK, while tobacco-free nicotine pouches are increasingly common across both the UK [56] and parts of Europe [57], often falling under different regulatory frameworks than vapes or combustible tobacco [58]. However, this is not consistent across Europe, with the Netherlands, Belgium, France and Germany banning the sale of nicotine pouches [58]. The findings therefore should not be taken to imply uniform effects across countries. Instead, they identify access-related mechanisms and patterns that may operate differently depending on local regulatory, market, and social conditions. Wider international comparison highlights the limits of generalising these findings beyond Europe. For example, Australia’s prescription-only model for e-cigarettes creates a distinct context for thinking about young people’s access [59]. In this setting, access to e-cigarettes is shifted towards illicitly imported and unregulated products or access to prescribed products through social networks [60]. Given the diversity of study designs, outcomes, and regulatory contexts, it was not feasible to stratify findings by regulatory environments within this review.

This review identifies several evidence gaps, pointing to areas for future research. For instance, while advertisements can influence acceptability [25], there were mixed findings on the effectiveness of advertising restrictions [36, 37], and no evidence on the effects of exposure to e-cigarette promotion via social media or influencer marketing on young people’s e-cigarette use. This gap is particularly notable given the rapid growth of social media and influencer marketing, which differ from traditional forms of advertising and may not be fully captured by existing regulatory approaches [61]. Similarly, though affordability has a known influence on young people’s e-cigarette use [26, 27, 31], the scarcity of European evidence on young people’s responses to e-cigarette price rises is a key evidence gap [62]. Without understanding how significantly price changes affect demand, it is difficult to anticipate how the new e-liquid tax in the UK will work in practice. Recent UK evidence-mapping work similarly identified major gaps in data on policy impacts, illicit markets and long-term health outcomes that constrain current policy modelling [62]. Further studies are therefore needed, especially considering new UK policies like the disposable vape ban [14] and e-liquid tax [15] whose real-world impacts will require evaluation. In addition, the high perceived accessibility of e-cigarettes by young people [26, 27, 31] suggests that retailer licensing regulations for e-cigarette sales will become increasingly important, for example to limit the proximity of retailers to schools, as with similar regulations in the UK regarding fast food outlets [63].

It is particularly interesting to map the five dimensions of access onto the potential UK regulatory changes for e-cigarettes that might be introduced under the Tobacco and Vapes Bill [16]. The powers within the Bill to regulate point-of-sale displays relate to acceptability; powers enabling stricter retailer controls (including licensing and enforcement) would influence accessibility; and the powers to make currently smoke-free places also vape-free would impact accommodation by restricting where people can use e-cigarettes. The ways in which these potential policy changes operate may be shaped by the recent ban on disposable e-cigarettes in the UK, potentially affecting availability and affordability (given that disposables tend to be cheaper products), and with the proposed new e-liquid tax, affecting affordability. This emphasises the need for a systems-thinking approach to understanding how these policy changes interact across the different dimensions of access.

In conclusion, the five dimensions of access provide a useful framework for thinking about how regulation may shape young people’s e-cigarette use within a complex system. This is particularly important due to the rapidly evolving e-cigarette market, the potential suite of forthcoming policy changes, and the diversity of young people’s methods of accessing and using e-cigarettes. Recognising the interactions between policies, markets, and social contexts may support more coordinated regulatory approaches aimed at limiting e-cigarette use among young people who do not smoke, while minimising unintended consequences. 

## Supplementary Information

Supplementary Material 1: PRISMA_2020_checklist.

Supplementary Material 2: Supplementary Methods.

Supplementary Material 3. Supplementary Results.

## Data Availability

All data generated or analysed during this study are included in this published article and its supplementary information files.

## Abbreviations

UK: United Kingdom
EU: European Union
WHO: World Health Organization
FCTC: Framework Convention on Tobacco Control
TPD: Tobacco Products Directive
MLSA: Minimum Legal Sales Age
COM-B: Capability, Opportunity, Motivation – Behaviour
JBI: Joanna Briggs Institute
PRISMA: Preferred Reporting Items for Systematic Reviews and Meta-Analyses
PROSPERO: International Prospective Register of Systematic Reviews
