# Supplementary material for: Access-related factors and e-cigarette use among 11–17-year-olds: a thematic synthesis of European studies using the five dimensions of access
Source: BMC Public Health. 2026 Feb 17;26:978. doi: 10.1186/s12889-026-26692-y (PMC13014995; doi:10.1186/s12889-026-26692-y)
Supplement: Supplementary file 2 — Supplementary Material 2: Supplementary Methods. [file 12889_2026_26692_MOESM2_ESM.docx]

**Search Strategy**

| **Concept** | **Terms** |
| --- | --- |
| Young people | adolescent* OR teenage* OR youth OR "young people" OR minors OR students OR "high school students" OR “secondary school students” OR child* OR “school-age child*” |
| E-cigarettes | e-cigarette* OR "electronic cigarettes" OR vaping " OR "e-cigs" OR "electronic nicotine delivery systems" OR “JUUL” OR “lost mary” OR “elf bar” OR “electronic smoking devices” OR vape* |
| Availability | availability OR presence OR supply OR "market presence" OR "product availability" OR retailers OR "store inventory" OR “supply side” OR “supply-side” OR supplier |
| Accessibility | accessibility OR proximity OR location OR distance OR "geographical access" OR "ease of access" OR "retail locations" OR store* OR shop* OR outlet* |
| Accommodation | accommodation OR "store hours" OR "sales policies" OR "delivery of sales" OR "store organisation" OR "sales practices" OR legislation OR underage OR “under-age” OR “under age” OR law OR laws OR regulation* |
| Affordability | affordability OR cost OR price OR "financial ability" OR "economic factors" OR "income level" OR "price sensitivity" OR tax OR duty |
| Acceptability | acceptability OR "social attitudes" OR "cultural attitudes" OR "peer influence" OR "public perceptions" OR "social acceptance" OR "cultural acceptance" OR “social norms” OR “peer use” OR culture OR “public opinion” OR perspective |

**Data Extraction Form**

- Title
- Authors
- Journal
- Type of Study
  - Quantitative
  - Qualitative
  - Mixed-methods
- Data
- Method
- Population
- Outcome Measures
- Policy Variables
- Study Results
- Country
- Year of Data Collection
- Dimensions of Access:
  - Availability
  - Accessibility
  - Affordability
  - Accommodation
  - Acceptability
- Policies:
  - Vape Free Spaces
  - Licensing
  - Price
  - Disposables Ban
  - Promotion
  - Age of Sale
- Limitations
- Author Comments
