# Supplementary material for: Access-related factors and e-cigarette use among 11–17-year-olds: a thematic synthesis of European studies using the five dimensions of access
Source: BMC Public Health. 2026 Feb 17;26:978. doi: 10.1186/s12889-026-26692-y (PMC13014995; doi:10.1186/s12889-026-26692-y)
Supplement: Supplementary file 3 — Supplementary Material 3. Supplementary Results. [file 12889_2026_26692_MOESM3_ESM.docx]

**Quality Appraisal**

| **Record Number** | **Study** | **Study Type** | **Methodological Strengths** |
| --- | --- | --- | --- |
| [24](file:///C:\Users\ecq17cdl\AppData\Local\Microsoft\Windows\INetCache\Content.MSO\21F54716.xlsx#RANGE!gid=0) | Maybe they should regulate them quite strictly until they know the true dangers': a focus group study exploring UK adolescents' views on e-cigarette regulation | Qualitative | Use of focus groups aligns well with the research question; and multiple researchers to enhance reliability. |
| [2](file:///C:\Users\ecq17cdl\AppData\Local\Microsoft\Windows\INetCache\Content.MSO\21F54716.xlsx#RANGE!gid=0)5 | Young people’s use of disposable vapes: A qualitative study | Qualitative | Use of multiple data collection methods: The study utilised a variety of methods (dyad-guided interviews, individual interviews, and group interviews) that helped to capture a comprehensive and naturalistic range of data, allowing participants to discuss topics in diverse formats. Involvement of participants in co-design: The use of co-designed prompt cards with input from young people (Patient and Public Involvement informants) strengthens the relevance and appropriateness of the questions asked. Systematic data analysis: Thematic analysis was done rigorously, with an inductive approach followed by deductive coding based on the Social Ecological Model. Verification of analysis through team discussions strengthens the robustness of the findings. |
| 26 | Youth’s engagement and perceptions of disposable e-cigarettes: a UK focus group study | Qualitative | Strong alignment between methodology and research question. Use of focus groups and online discussions to capture diverse perspectives. Thematic analysis ensures in-depth interpretation. Participant voices are well represented. |
| 27 | Use of tobacco and e-cigarettes among youth in Great Britain in 2022: Analysis of a cross-sectional survey | Analytical Cross-Sectional | Use of the ASH/YouGov national dataset provides a large, representative sample. Clearly defined inclusion criteria. Detailed description of study population and setting. Valid and reliable measures for exposure and outcomes. Use of multivariate regression to adjust for confounders. |
| 28 | Impacts of EU Tobacco Products Directive regulations on use of e-cigarettes in adolescents in Great Britain: a natural experiment evaluation | Quasi-Experimental Studies | Robust quasi-experimental design (ITS) is ideal for policy evaluation. Uses a large, nationally representative sample across multiple years. Sophisticated multilevel statistical analysis controls for key confounders and data clustering. Use of smoking as a 'control outcome' adds specificity to the findings. |
| 29 | Co-production of a youth advocacy video on the harms of e-cigarette advertising in Scotland | Qualitative | Clear alignment between the research question and methodology; thematic analysis well-executed; ethical approval granted. |
| 30 | Teenage perceptions of electronic cigarettes in Scottish tobacco-education school interventions: co-production and innovative engagement through a pop-up radio project | Qualitative | Strong alignment between methodology and research question, with participant-led responses capturing subjective perceptions. Participants’ voices well represented. |
| 31 | Changing awareness and sources of tobacco and e-cigarettes among children and adolescents in Great Britain | Analytical Cross-Sectional | Large, representative sample, use of logistic regression models to control for confounders, repeated annual survey method enhancing reliability, sensitivity analysis to check robustness, clear definition of exposure and outcome. |
| 32 | Young people's perspectives of e-cigarette use in the home | Qualitative | The qualitative methodology is well-matched to the research question and objectives, ensuring an in-depth exploration of young people’s perceptions of e-cigarette use in home environments. Thematic analysis aligns with the interpretivist approach, and the use of in-depth interviews allows for a rich understanding of participants’ lived experiences. The study includes direct quotes and participant perspectives, enhancing transparency and credibility. The conclusions are directly linked to the data analysis and interpretation, ensuring that findings are grounded in participant accounts rather than speculative generalisations. |
| 33 | Do stronger school smoking policies make a difference? Analysis of the health behaviour in school-aged children survey | Analytical Cross-Sectional | Clearly defined inclusion criteria. Detailed description of study population and setting. Valid and reliable measures for exposure and outcomes. Use of multivariate regression to adjust for confounders. |
| 34 | Relationship between e-cigarette point of sale recall and e-cigarette use in secondary school children: a cross-sectional study | Analytical Cross-Sectional | Clear sample criteria, exposure and outcomes measured using standard, validated methods. Confounders identified and adjusted for using multivariate regression; appropriate statistical analysis with missing data handling. |
| 35 | E-cigarette use and conventional cigarette smoking among European students: findings from the 2019 ESPAD survey | Analytical Cross-Sectional | Clear inclusion criteria with a large, diverse sample from 35 countries, use of validated ESPAD methodology for measuring substance use, identification of key confounders, multivariate regression and random-slope models to adjust for confounding factors, widely used reliable outcome measures. |
| 36 | Cross-sectional study of the associations between the implementation of the WHO FCTC tobacco advertising, promotion and sponsorship bans and current e-cigarette use among youth from countries with different income levels | Analytical Cross-Sectional | The study clearly defines its sample (adolescents aged 11–17 in schools) and uses a structured sampling method (GYTS two-stage cluster sampling). Uses objective data from the WHO FCTC Implementation Database and a standardised survey (GYTS) for e-cigarette use. Uses multilevel logistic regression, adjusted models, and stratified analyses, demonstrating a robust analytical approach. |
| 37 | Association between the implementation of tobacco control policies and adolescent vaping in 44 lower-middle, upper-middle, and high-income countries | Analytical Cross-Sectional | Use of a well-established survey (GYTS), mixed effect logistic regression to control for individual and country-level confounders, inclusion of robustness checks and sensitivity analyses, large sample size from diverse countries. |
| 38 | How are Adolescents Getting Their Vaping Products? Findings from the International Tobacco Control (ITC) Youth Tobacco and Vaping Survey | Analytical Cross-Sectional | Clear inclusion criteria, sample described in detail. Exposure measured using self-reported methods with comparison to other national surveys for validity, confounders identified and adjusted for using multivariate regression. |
| 3[9](file:///C:\Users\ecq17cdl\AppData\Local\Microsoft\Windows\INetCache\Content.MSO\21F54716.xlsx#RANGE!gid=1785883975) | Electronic cigarette advertising and teen smoking initiation | Analytical Cross-Sectional | The study clearly defines inclusion criteria, such as age range (12-17 years) and school type. The study provides comprehensive details on participant characteristics, including sociodemographic factors like age, gender, migration background, and socioeconomic status. Exposure to e-cigarette advertisements was measured using reliable, externally sourced data (from a media monitoring agency), which adds credibility to the exposure measurement. Multiple potential confounders (e.g., sociodemographic factors, baseline behaviours) were identified and controlled for through statistical adjustment. |
| 40 | Temporal changes and correlates of tobacco and E-cigarettes use among school-going students in Albania: insights from global youth tobacco surveys (2015–2020) | Analytical Cross-Sectional | The study uses the GYTS, which implies inclusion criteria. The use of the GYTS ensures standardised, objective criteria for assessing e-cigarette use, supporting validity. The study controls for age and sex in its statistical analysis, using logistic regression, which is an appropriate method for this type of study. The study employs descriptive statistics, chi-square tests for associations, and logistic regression for temporal trends. |
| 41 | Youth Access to Electronic Cigarettes in an Unrestricted Market: A Cross-Sectional Study from Poland | Analytical Cross-Sectional | Clearly defines inclusion criteria, provides demographic details and context of the unregulated e-cigarette market in Poland. Defines access methods using standard categories. |
| 42 | Impact of an Outdoor Smoking Ban at Secondary Schools on Cigarettes, E-Cigarettes and Water Pipe Use among Adolescents: An 18-Month Follow-Up | Quasi-Experimental Studies | Control group design, multiple follow-up measurements (pre- and post-intervention), robust statistical analyses (e.g., multilevel analysis, Bayesian estimation for missing data), consideration of confounding factors, use of consistent measurement methods across groups. |
| 43 | E-cigarette use in global digital youth culture. A qualitative study of the social practices and meaning of vaping among 15–20-year-olds in Denmark, Finland, and Norway | Qualitative | The cross-national approach is a significant strength. Comparing different regulatory environments allows for valuable insights into the impact of policy variations on young people's vaping behaviours. The level of detail that the focus groups went into provides rich, nuanced data on young people's perceptions and experiences with e-cigarettes. The involvement of multiple researchers from different countries can enhance objectivity and reduce bias in the analysis. |
| [44](file:///C:\Users\ecq17cdl\AppData\Local\Microsoft\Windows\INetCache\Content.MSO\21F54716.xlsx#RANGE!gid=1102429603) | ‘Vaping and fidget-spinners’: A qualitative, longitudinal study of e-cigarettes in adolescence | Qualitative | Clearly grounded in Actor-Network Theory (ANT) and Interaction Ritual Theory (IRT). Methodology aligns well with research aims. Uses semi-structured interviews and thematic analysis effectively. Detailed analysis, considering time and trend aspects of e-cigarette perceptions |

| **Record Number** |  | **Key Limitations** | **Overall Quality Assessment** |
| --- | --- | --- | --- |
| [24](file:///C:\Users\ecq17cdl\AppData\Local\Microsoft\Windows\INetCache\Content.MSO\21F54716.xlsx#RANGE!gid=0) | Maybe they should regulate them quite strictly until they know the true dangers': a focus group study exploring UK adolescents' views on e-cigarette regulation | No explicit discussion of researcher positionality or reflexivity. Does not state a philosophical perspective. | Moderate-High |
| [2](file:///C:\Users\ecq17cdl\AppData\Local\Microsoft\Windows\INetCache\Content.MSO\21F54716.xlsx#RANGE!gid=2057935511)5 | Young people’s use of disposable vapes: A qualitative study | Lack of explicit researcher reflexivity: There is no clear discussion on the researcher's positionality or how their own perspectives might influence the research process, potentially affecting objectivity. Limited methodological discussion: Although the methodology is robust, the study does not explicitly state the philosophical or theoretical orientation guiding the research, which could limit the transparency of the approach. | Moderate-High |
| 26 | Youth’s engagement and perceptions of disposable e-cigarettes: a UK focus group study | No explicit statement of researcher positionality or influence. Lack of discussion on reflexivity. | Moderate-High |
| 27 | Use of tobacco and e-cigarettes among youth in Great Britain in 2022: Analysis of a cross-sectional survey | Reliance on self-reported data, which may introduce bias (e.g., social desirability or recall bias). Potential confusion about what constitutes an e-cigarette. | High |
| 28 | Impacts of EU Tobacco Products Directive regulations on use of e-cigarettes in adolescents in Great Britain: a natural experiment evaluation | Relies on self-reported data for smoking/vaping without biochemical validation. As a natural experiment, it cannot definitively rule out all other co-occurring events that may have influenced the outcome. Only two data points were available post-intervention to model the new trend. | High |
| 29 | Co-production of a youth advocacy video on the harms of e-cigarette advertising in Scotland | Lack of explicit discussion of reflexivity; minimal detail on the researcher's positionality; limited justification for chosen methodology. | Moderate-High |
| 30 | Teenage perceptions of electronic cigarettes in Scottish tobacco-education school interventions: co-production and innovative engagement through a pop-up radio project | No explicit statement on researcher positionality or reflexivity. Lack of deeper or more structured analysis limits depth of findings, doesn't address researcher influence. | Moderate-High |
| 31 | Changing awareness and sources of tobacco and e-cigarettes among children and adolescents in Great Britain | Since participants could see the different e-cigarette packaging designs, they were aware of their assigned condition, introducing the potential for bias in responses. | Moderate-High |
| 32 | Young people's perspectives of e-cigarette use in the home | Self-reported data may introduce bias, though steps are taken to minimise this. No comparison to objective measures for exposure. | High |
| 33 | Do stronger school smoking policies make a difference? Analysis of the health behaviour in school-aged children survey | The study does not include a statement locating the researcher’s cultural or theoretical positioning, nor does it discuss how the researcher’s background or biases may have influenced the research process. The study does not critically reflect on how the researcher may have shaped data collection, analysis, or interpretation. While the study follows an interpretivist approach, it does not explicitly state its philosophical perspective, making it slightly unclear how methodological choices are underpinned by broader epistemological or theoretical frameworks. | Moderate-High |
| [34](file:///C:\Users\ecq17cdl\AppData\Local\Microsoft\Windows\INetCache\Content.MSO\21F54716.xlsx#RANGE!gid=493164903) | Relationship between e-cigarette point of sale recall and e-cigarette use in secondary school children: a cross-sectional study | Reliance on self-reported data, which may introduce bias (e.g., social desirability or recall bias). | High |
| 35 | E-cigarette use and conventional cigarette smoking among European students: findings from the 2019 ESPAD survey | Self-reported data may introduce recall and social desirability biases. No evidence of intra- or inter-observer reliability for exposure measures. Assumptions in handling missing data could affect results. | High |
| 36 | Cross-sectional study of the associations between the implementation of the WHO FCTC tobacco advertising, promotion and sponsorship bans and current e-cigarette use among youth from countries with different income levels | Reliance on self-reported data introduces potential for reporting biases (e.g., over- or under-reporting); no mention of specific training or education for data collectors. | High |
| 37 | Association between the implementation of tobacco control policies and adolescent vaping in 44 lower-middle, upper-middle, and high-income countries | While GYTS is a standardised survey, self-reported e-cigarette use may introduce recall or social desirability biases. The study could have provided further breakdowns (e.g., gender, school type) to enhance specificity. | High |
| 38 | How are adolescents getting their vaping products? Findings from the international tobacco control (ITC) youth tobacco and vaping survey | Self-reported data may be subject to recall and social desirability biases; lack of standardisation in definitions of “vaping product” and “vaping” across respondents; no measurement of socio-economic status (SES) as a confounder. | Moderate-High |
| 39 | Electronic cigarette advertising and teen smoking initiation | The outcome of e-cigarette initiation was self-reported, which can introduce bias, such as social desirability bias or recall bias. The study does not provide sufficient detail on the assumptions underlying the statistical models used, which could affect the validity of the results. The study did not explicitly mention exclusion criteria, which could have clarified the sampling process. | Moderate-High |
| 40 | Temporal changes and correlates of tobacco and E-cigarettes use among school-going students in Albania: insights from global youth tobacco surveys (2015–2020) | While age and sex were adjusted for, other potential confounders like socioeconomic status or school grade were only included descriptively, leaving uncertainty about their impact on the results.  As with most surveys of this type, self-reported data could introduce bias (over- or under-reporting), though the large sample size and standardised protocols help mitigate this risk. | Moderate-High |
| 41 | Youth access to electronic cigarettes in an unrestricted market: a cross-sectional study from Poland | Reliance on self-reported data with unclear validation of survey tools; no explicit strategies for dealing with confounders; lacks regression analysis to adjust for confounding factors. | Low-Moderate |
| 42 | Impact of an outdoor smoking ban at secondary schools on cigarettes, e-cigarettes and water pipe use among adolescents: an 18-month follow-up | Missing data could potentially affect follow-up results, and the control group was not randomly assigned, which introduces potential bias. The study lacks detailed analysis of how both groups were treated outside the intervention. | Moderate-High |
| 43 | E-cigarette use in global digital youth culture. A qualitative study of the social practices and meaning of vaping among 15–20-year-olds in Denmark, Finland, and Norway | Variations in the composition of the samples across countries, particularly the proportion of current and former e-cigarette users, could introduce bias. While the researchers state that results were largely similar, the potential impact of these differences should be considered. Additionally another challenge related to the samples is the difficulty in recruiting participants due to low vaping prevalence limits the generalisability of the findings. The results may not be representative of populations with higher vaping rates. | High |
| 44 | ‘Vaping and fidget-spinners’: A qualitative, longitudinal study of e-cigarettes in adolescence | No discussion of researcher reflexivity or influence on findings. Does not explicitly position the researcher culturally or theoretically. | High |
